# Noise Reduction for a Virtual Grid Using a Generative Adversarial Network in Breast X-ray Images

**DOI:** 10.3390/jimaging9120272

**Published:** 2023-12-07

**Authors:** Sewon Lim, Hayun Nam, Hyemin Shin, Sein Jeong, Kyuseok Kim, Youngjin Lee

**Affiliations:** 1 Department of Health Science, General Graduate School of Gachon University, 191, Hambakmoe-ro, Yeonsu-gu, Incheon 21936, Republic of Korea; tpdnjs728@gachon.ac.kr; 2 Department of Radiological Science, Gachon University, 191, Hambakmoe-ro, Yeonsu-gu, Incheon 21936, Republic of Korea; lime525@gachon.ac.kr (H.N.); shm0149@gachon.ac.kr (H.S.); tpdls2001@gachon.ac.kr (S.J.); 3 Department of Biomedical Engineering, Eulji University, 533, Sanseong-daero, Sujung-gu, Seongnam-si 13135, Republic of Korea

**Keywords:** virtual grid, breast X-ray image, noise reduction, generative adversarial network, quantitative evaluation of image quality

## Abstract

In this study, we aimed to address the issue of noise amplification after scatter correction when using a virtual grid in breast X-ray images. To achieve this, we suggested an algorithm for estimating noise level and developed a noise reduction algorithm based on generative adversarial networks (GANs). Synthetic scatter in breast X-ray images were collected using Sizgraphy equipment and scatter correction was performed using dedicated software. After scatter correction, we determined the level of noise using noise-level function plots and trained a GAN using 42 noise combinations. Subsequently, we obtained the resulting images and quantitatively evaluated their quality by measuring the contrast-to-noise ratio (CNR), coefficient of variance (COV), and normalized noise–power spectrum (NNPS). The evaluation revealed an improvement in the CNR by approximately 2.80%, an enhancement in the COV by 12.50%, and an overall improvement in the NNPS across all frequency ranges. In conclusion, the application of our GAN-based noise reduction algorithm effectively reduced noise and demonstrated the acquisition of improved-quality breast X-ray images.

## 1. Introduction

Scattered radiation obscures tissue structures in X-ray images, thereby diminishing diagnostic precision. Physical grids are used to mitigate such scattered radiation. However, a physical grid induces a cut-off phenomenon, and increases the patient’s radiation dose [1]. In particular, the use of physical grids under various imaging conditions leads to an increase in retakes and exposure time due to patient motion [2]. Software-based virtual grid methods have been proposed to address these issues associated with physical grids [3,4,5]. However, when using virtual grids, there is the problem of noise amplification after scatter removal, which is represented using the following formula [6]:

(1) $$\frac{Variance(n_{sca})}{Variance(n_{non-sca})}\approx \;{\left(1+\frac{S}{P}\right)}^{2}$$

where $n_{sca}$ represents the residual noise component after scattering correction, $n_{non-sca}$ signifies the noise component without scattering correction, S stands for the pure scattering noise component, and P denotes the noise-free primary signal. Therefore, an issue arises when noise is amplified after scattering correction, leading to a degradation in the contrast-to-noise ratio (CNR) of the images.

Noise amplification is a significant factor that degrades the overall image quality in X-ray imaging [6,7]. In breast X-ray imaging, noise reduces the efficiency of detecting microcalcifications, making it difficult to distinguish them from benign micro tumors, thereby compromising diagnostic accuracy [8]. Breast tissue is densely composed, which makes it challenging to detect microcalcifications. In addition, the presence of noise further reduces the diagnostic accuracy of early breast cancer [9].

To effectively reduce this noise, it is essential to employ noise reduction techniques and also estimate the noise level in the image. Since low radiation doses are used in breast examinations, the proportion of quantum noise increases compared to other types of examinations. The use of filters that are unsuitable for this noise level result in inadequate improvement in the image quality. Various techniques exist to estimate the noise level function (NLF), including patch-based approaches [10], statistical approaches, and variance-stabilizing transforms [11]. The patch-based approach involves subdividing an image into small patches and selecting the patches with an intensity standard deviation close to the minimum values. Subsequently, noise levels are estimated within the chosen patches. However, this method tends to overestimate low noise levels and underestimate high noise levels. To address this issue, a statistical approach has been proposed that observes changes in kurtosis due to noise and utilizes this effect to estimate the noise levels. Variance-stabilizing transforms refer to transformation methods that stabilize the variance of data, allowing for the accurate estimation and analysis of noise characteristics by stabilizing the noise level. However, these techniques require prior information regarding the noise parameters. Moreover, estimating the NLF from complex images with Poisson–Gaussian mixture noise is challenging [12]. Consequently, a noise parameter estimation method based on non-parametric detection in homogeneous blocks using Kendall’s τ coefficient for estimating the NLF in images with Poisson–Gaussian mixture noise has been proposed [13,14,15]. This method estimates a polynomial NLF based on N1-norm approximations using the relationship between mean and variance in homogeneous regions.

Therefore, in this study, we aimed to quantify the noise level quantitatively using a Poisson–Gaussian mixture model to represent the radiation noise. Subsequently, we divided the acquired images into small patches and extracted the homogeneous patches using the Kendall Tau method-based rank correlation coefficient. By plotting based on mean and variance, the first-degree trend line was calculated to determine the final noise level. Furthermore, we planned to perform deep learning training based on Generative Adversarial Networks (GANs) [16] using a noise-level-specific database and develop a dose-specific and customized noise reduction method.

GANs are neural networks in which a generator and a discriminator engage in adversarial training. During the training of GANs, the generator is trained to produce samples that match the distribution of real data. Consequently, the generator creates synthetic data that resembles real data, and the discriminator is trained to distinguish between real and fake data [17]. In this study, we leverage this characteristic of GANs to generate noisy images. GANs exhibit a tendency to produce results that are closer to reality in comparison to other network models, which led us to believe that they can generate more naturally noisy images. We also anticipated that GANs would enable the generation of high-quality images for tasks involving image synthesis. Furthermore, by employing two networks, the generator and the discriminator, in the training process, GANs enhance the network stability through competition between these two networks. This competitive framework enables rapid and effective learning, which motivated us to choose GANs for this research.

Thus, the objective of this study was to model an algorithm that can estimate radiation noise at various noise levels to address the issue of noise amplification when using virtual grids. We intended to build an improved virtual grid software by developing a noise reduction algorithm based on GANs.

## 2. Materials and Methods

### 2.1. Compliance with Ethical Standards

This study was conducted in accordance with the tenets of the Declaration of Helsinki after obtaining approval from the Institutional Review Board (1041849-201908-BM-119-01). Written informed consent was obtained from all the patients before they were included in the study.

### 2.2. Acquisition of Breast X-ray Images

**Figure 1:**
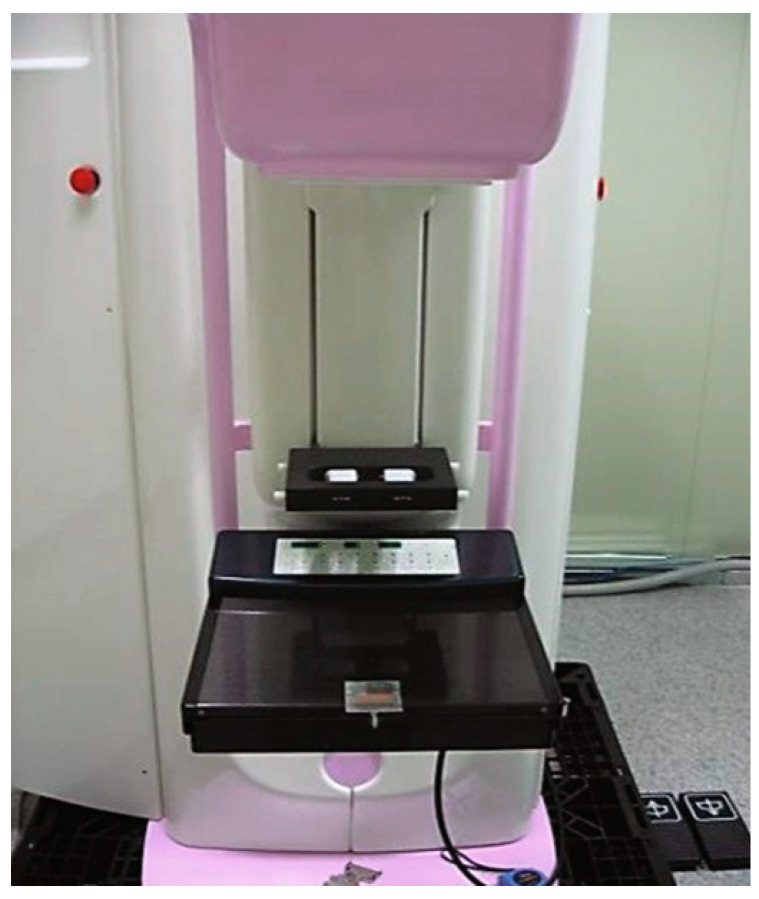
Photograph of the mammographic system used in the experiment.

Breast X-ray images were acquired using a Sizgraphy device (Rayence Co., Ltd., Hwaseong-si, Republic of Korea) with dimensions of 3300 × 2432 (Figure 1). The equipment comprised an X-ray generator with settings of 28 kVp and 40 mA, along with a flat-panel detector of CMOS type with a pixel size of 70 μm.

### 2.3. Scatter Correction Using a Virtual Grid

**Figure 2:**
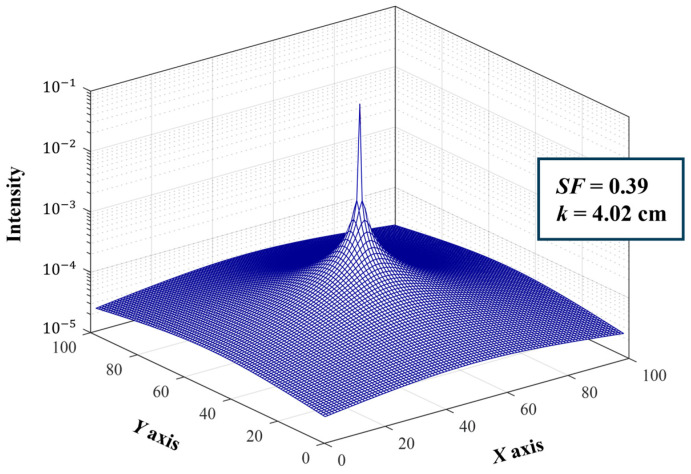
2D sPSF used in the simulation to generate scattering images. The parameters used in the sPSF were SF = 0.39 and k = 4.02 cm.

The two-dimensional (2D) scatter point spread function (sPSF) illustrates how a point source of light is ambiguously represented due to scattering. The 2D sPSF is used to describe the ambiguity caused by scattering, as scattered light disperses in various directions when passing through an optical system, causing the point source of light to spread more widely, resulting in image blurring [18]. The 2D sPSF represents the intensity of the PSF in x and y coordinates, depicting how this light scatters and diffuses ambiguously, thereby generating a certain shape around the light source. In this study, a 2D sPSF was utilized with scatter fraction (SF) = 0.39 and scatter range (k) = 4.02 to simulate the scattering component by introducing artificial scattering into the acquired breast X-ray images. The values of SF and k were estimated by measuring the log contrast ratio on a PMMA phantom by Ducote et al. The expression for sPSF is as follows [19]:

(2) $$sPSF(\gamma )=\frac{\delta (\gamma )}{\gamma }+\frac{SF}{(1-SF)2kr}e^{-\gamma /k}$$

(3) $$\delta (\gamma )=\left\{\begin{array}{c} \infty ,\;\;\gamma =0\\ 0,\;\;\;\gamma \neq 0 \end{array}\right.$$

(4) $$\int_{-\infty }^{\infty }\delta \left(\gamma \right)dx=1$$

where γ represents the radial distance and δ(γ) denotes the delta function representing the first-order component. Figure 2 shows the sPSF configuration used to simulate the scatter images.

**Figure 3:**
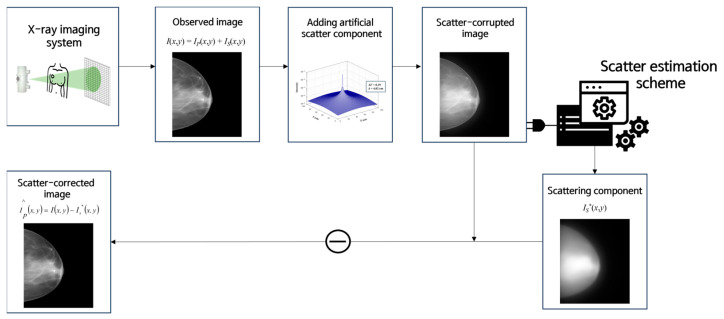
Simplified diagram of scatter correction that subtracts the scatter component from the observed image after acquiring an X-ray image to obtain a scatter-corrected image.

After artificially introducing the scatter component, scatter correction was performed using a virtual grid software. Using the virtual grid software, the scatter component in the image was estimated and subtracted from the original image to obtain a scatter-corrected image. A schematic representation of the scatter correction process is shown in Figure 3 [20].

**Figure 4:**
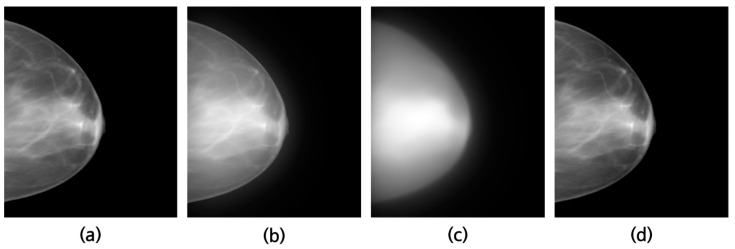
An example of the (**a**) exact, (**b**) scatter-corrupted, (**c**) scattering, and (**d**) scatter-corrected image.

Figure 4a represents an image without scatter, while (b) depicts an image with scatter introduced using the sPSF. Figure 4c shows the image of the estimated scatter component from the virtual grid software, and (d) illustrates the scatter-corrected image obtained by subtracting (c) from (b).

### 2.4. Noise Suppression Using GANs

#### 2.4.1. Noise Level Quantification

After performing scatter correction using a virtual grid software, a Poisson–Gaussian mixture model was utilized to analyze the noise characteristics of breast X-ray images. The equation is as follows [12]:

(5) y=x+η(x)ε

where y represents the X-ray images, x is the noise-free original image, η(x) is the standard deviation of noise distribution, and ε denotes the Gaussian noise. In this model, the noise variance $\eta^{2}(x)$ can be separated into Poisson and Gaussian components, and expressed using the following equation [12]:

(6) $$\eta^{2}(x)=\alpha x+\beta^{2}$$

where α and β denote the standard deviations of Poisson and Gaussian noise, respectively. In this study, noise level was estimated using NLF proposed by Sutour et al. [13]. First, the noisy image was divided into 16 × 16 pixel-sized squares and regions with weak signal-to-noise ratios were detected using Kendall’s τ-coefficient. In these homogeneous blocks, where the signal variation is minimal compared to the noise, noise estimation becomes feasible. Therefore, NLF can be estimated for each block.

#### 2.4.2. GAN Training

**Figure 5:**
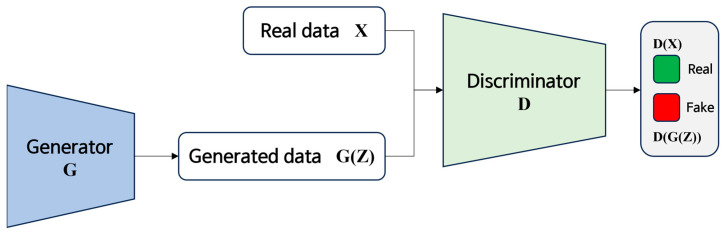
Illustration of GAN architecture used in this study.

The architecture of the GAN used for training is illustrated in Figure 5. The autoencoder focuses on reconstructing the training data itself, but the encoder–decoder architecture is primarily employed for generative model tasks by encoding input data into a specific representation and subsequently restoring this representation back to the original data through a decoder. Therefore, we have utilized an encoder–decoder structure in our approach, as our emphasis lies in the task of generating noisy images. The parameters for training the GAN are listed in Table 1. A combination of 42 noise levels, estimated through NLF, was applied to the breast X-ray images and used as input data. Original breast X-ray images, which were the images before the noise was added, were subtracted from the noisy breast X-ray images to serve as label data during training. For training, the original images were interpolated using the bilinear method to make it 4950 × 3648, and the image was sequentially divided into 256 × 256 patches, starting at position (0, 0) and setting stride to 256. In total, 54,686 training patches, 6100 validation patches, and 2128 test patches were used.

### 2.5. Quantitative Evaluation of the Noise-Reduced Image

To quantitatively assess noise reduction in the image resulting from subtracting the output image trained by the GAN from the input image, the following metrics were measured: CNR, coefficient of variation (COV), and normalized noise power spectrum (NNPS).

#### 2.5.1. CNR and COV

CNR represents the contrast of regions of interest (ROI) with respect to noise. Higher CNR values indicate better image quality. The formula used for CNR is as follows [21]:

(7) $$CNR=\frac{\left|S_{A}-S_{B}\right|}{\sqrt{\sigma_{A}^{2}+\sigma_{B}^{2}}}$$

where $S_{A}$ and $\sigma_{A}$ represent the mean and standard deviation of signal intensity within the ROI, respectively, while $S_{B}$ and $\sigma_{B}$ represent the mean and standard deviation of signal intensity within the background region, respectively.

COV represents the relative range distribution of pixel values, and is calculated by dividing the standard deviation by the mean. It provides a quantitative measure with statistical significance, enabling joint analysis of signal and noise. A smaller COV value indicates lower noise in the image, and the formula used is as follows [22]:

(8) $$COV=\frac{\sigma_{A}}{S_{A}}$$

#### 2.5.2. NNPS

The noise power spectrum (NPS) describes the spatial frequency dependency of noise and is explained by the Wiener spectrum [23]. The noise characteristics in an image are quantified using the NNPS, which represents the average area (in mm^2^) occupied by individual photons per unit area. The formula for NNPS is as follows [24]:

$$NPS=\underset{N_{x},N_{y}\to \infty }{\lim}\left(N_{x}N_{y}\Delta x\Delta y\right)<{\left|{FT}_{nk}I\left(x,y\right)-S\left(x,y\right)\right|}^{2}\;=\underset{N_{x},N_{y}\to \infty }{\lim}\underset{M\to \infty }{\lim}\frac{\left(N_{x}N_{y}\Delta x\Delta y\right)}{M}\sum_{m=1}^{M}{\left|{FT}_{nk}I\left(x,y\right)-S\left(x,y\right)\right|}^{2}\;=\underset{N_{x},N_{y},M\to \infty }{\lim}\frac{\Delta x\Delta y}{M\cdot N_{x}N_{y}}\sum_{m=1}^{M}\langle {\left|\sum_{i=1}^{N_{x}}\sum_{j=1}^{N_{y}}\left(I\left(x_{i},y_{j}\right)-S(x_{i},y_{j})\right)e^{-2\pi i(u_{n}x_{i}+v_{k}y_{i})}\right|}^{2}\rangle$$

(9) $${NPS}_{normalized}\left(u,v\right)=\frac{NPS(u,v)}{{\left(large\;area\;signal\right)}^{2}}$$

where I(x,y) denotes the average image intensity, S(x,y) is the average background intensity, $N_{x}$, $N_{y}$ denotes the pixel numbers along the X- and Y-axes, and Δx, Δy represents the pixel sizes along the X- and Y-axes, respectively. NNPS is a function of spatial frequency that estimates variations in noise amplitude, providing information about the noise and spatial resolution characteristics of the image.

## 3. Results

### 3.1. Training Results Image

**Figure 6:**
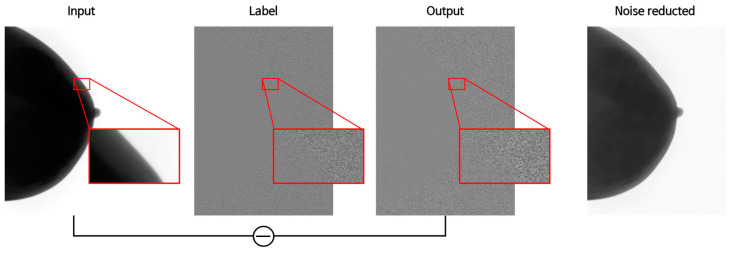
Diagram depicting input, label, output and the noise reducted image. A portion of input, label, and output images were magnified.

After training on patches measuring 256 × 256, the process involved stitching these patches back into the original size. Following training, testing was performed to obtain the output image, which was then subtracted from the input image to ultimately obtain the noise-reduced image (Figure 6).

### 3.2. Quantitative Evaluation Results

#### 3.2.1. CNR and COV Measurement Results

**Figure 7:**
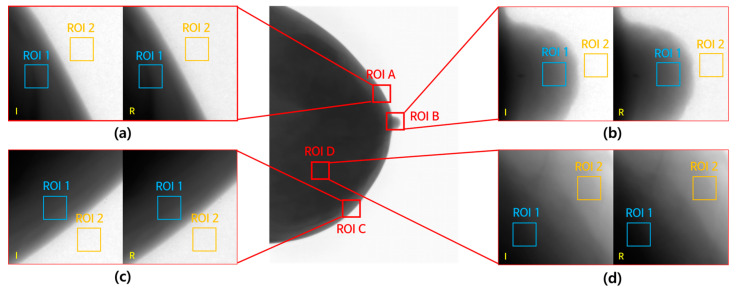
The (**a**) upper breast, (**b**) nipple, (**c**) lower breast, and (**d**) internal tissue of the breast ROIs for CNR and COV measurements.

Figure 7 shows the enlarged ROIs used to measure the CNR and COV. CNR values were measured for each ROI, from ROI A to ROI D, within both ROI1 and ROI2, and the COV values were measured within ROI1. ROI A represents the upper part of the breast, ROI B the nipple area, ROI C the lower part of the breast, and ROI D the internal tissue within the breast. In ROI D, the aim was to compare the areas with high and low densities within the breast tissue.

**Figure 8:**
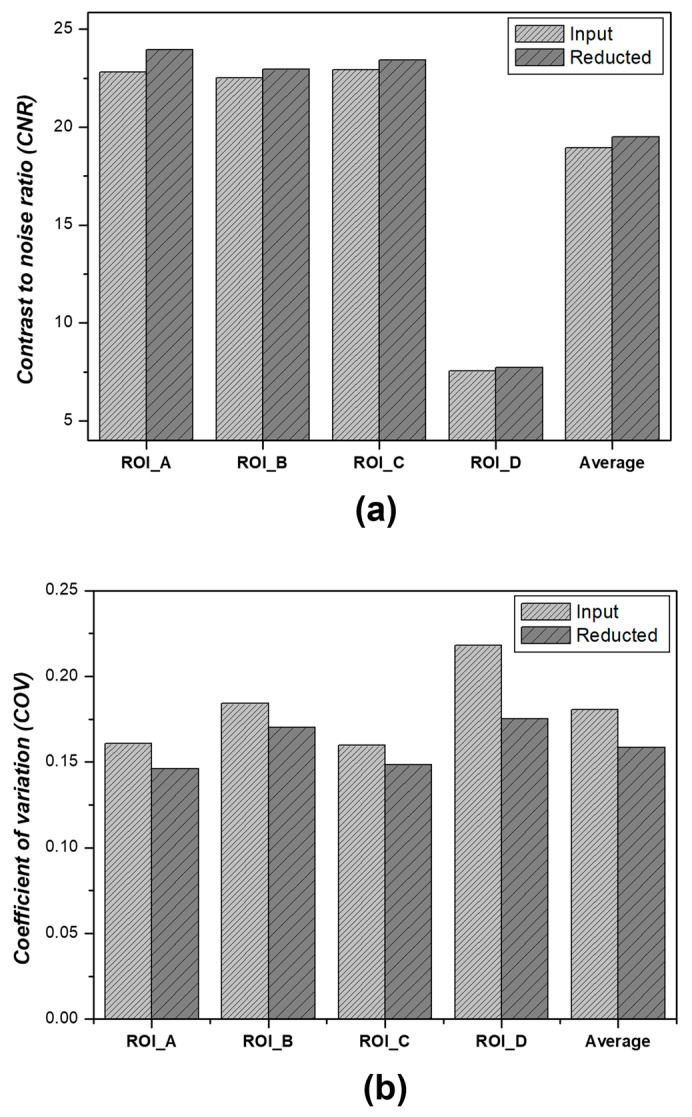
Results showing measurements for ROI A, ROI B, ROI C, ROI D, and the average (**a**) CNR and (**b**) COV.

The CNR and COV measurement results for each ROI are shown in Figure 8. We measured the CNR and COV for both input and denoised images in each of the regions of interest (ROI A, ROI B, ROI C, and ROI D) and represented the results graphically.

In the context of the CNR, before applying the algorithm, the values in ROI A, ROI B, ROI C, and ROI D were approximately 22.814, 22.512, 22.932, and 7.545, respectively. When the algorithm developed using GAN was applied, the CNR values in ROI A, ROI B, ROI C, and ROI D were approximately 23.952, 22.948, 23.422, and 7.714, respectively, indicating an average improvement of 2.80%. Before applying the algorithm, the COVs in ROI A, ROI B, ROI C, and ROI D were approximately 0.160, 0.184, 0.159, and 0.218, respectively. After applying the algorithm, they were measured to be 0.146, 0.170, 0.148, and 0.175, respectively, showing an average improvement of 12.50%.

#### 3.2.2. NNPS Measurement Results

**Figure 9:**
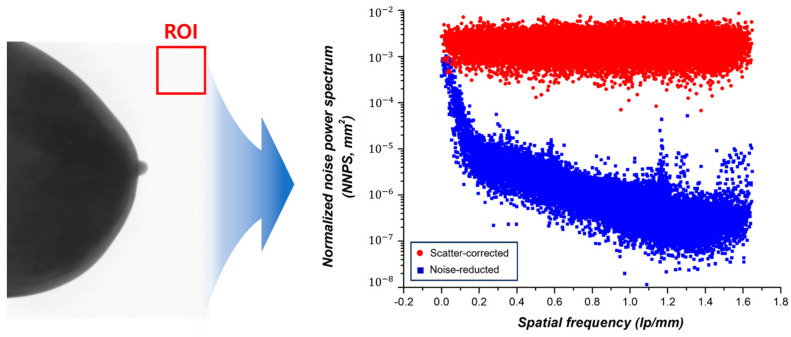
Illustration of ROI for NNPS measurement and a graph depicting the measured NNPS.

To analyze the noise and spatial resolution characteristics of the image by estimating the noise amplitude variations, the NNPS was measured. The measurement results are shown in Figure 9. To measure subtle noise fluctuations, we established an ROI, as depicted in Figure 9, given that it is imperative to conduct measurements in an area characterized by minimal signal variability within the background. The NNPS measurement results confirmed that noise reduction led to improvement in the spatial resolution characteristics of the image across all frequency ranges.

## 4. Discussion

Scattered radiation degrades the quality of X-ray images and obscures the boundaries and structures of objects, thereby diminishing the clarity of tissue structures and reducing diagnostic accuracy [25]. Grids are employed to mitigate such scattered radiation. However, the use of moving grids, particularly those designed to eliminate grid lines visible in the images, result in an increase in patient’s entrance skin exposure [26]. This is because, during X-ray exposure, continuous blocking due to lateral decentering occurs, while the grid moves back and forth by 1–3 cm, leading to the loss of primary radiation. A loss rate exceeding 20% necessitates an increase in X-ray exposure to compensate for this loss. Furthermore, when using moving grids, the X-ray photons are evenly dispersed in the image, which means that, for a given unit area, the incident photon quantity is the same as that when using a stationary grid. However, the use of moving grids results in an approximately 15% decrease in radiographic density in comparison to the use of a stationary grid, which in turn necessitates an increase in X-ray exposure to compensate for this reduction. Consequently, patient entrance skin exposure also increases [27].

Additionally, the use of physical grids increases the likelihood of the cut-off phenomenon, which leads to a deterioration in image quality. The cut-off phenomenon occurs when the incident direction of X-rays does not align with the grid, resulting in the primary radiation not reaching the detector or magnifying the grid lines. If grid line magnification occurs, the image fails to form, displaying only the transparent regions. Cut-off phenomena in grids can be primarily categorized into four scenarios: first, when the grid’s front and back sides are reversed; second, when there is a misalignment due to the lateral displacement of the grid’s center; third, when the distance between the focus and grid is inappropriate; and fourth, when a combination of the lateral displacement of the grid’s center and an improper distance between the focus and grid is present [27]. Therefore, physical grids have various physical limitations. The use of virtual grids is one approach to overcome these challenges. However, the use of virtual grids also has its limitations. Virtual grids effectively remove scattered radiation, but amplify the overall noise in the images.

Research addressing noise issues in medical imaging is ongoing. Typical noise in medical imaging follows a Poisson–Gaussian distribution, and various filtering algorithms have been developed to remove such noise. These include filters such as the Gaussian filter, median filter, Wiener filter, fusion filter, and median-modified Wiener filter, all of which aim to overcome the drawbacks of traditional local filters [28]. Additionally, noise removal techniques based on wavelet transforms and deep learning have also been proposed [29].

Deep learning is widely used in medical imaging for diagnosis, surgical planning, patient prognosis, and new approaches to treatment [30]. In particular, active research is being conducted in the fields of image segmentation and diagnosis [31]. However, to improve the accuracy of deep-learning-based image segmentation and diagnosis, noise reduction is imperative as a preprocessing step [32]. Furthermore, when conducting training using deep learning, training should be performed with a noise-reduced dataset to enhance prediction accuracy and facilitate effective network optimization. Consequently, there is a growing need for deep learning networks to address noise problems. In contrast to the diverse developments in deep learning networks, research on deep-learning-based noise reduction to address noise issues caused by virtual grid usage is relatively underdeveloped.

A GAN is a deep learning network consisting of a generator and a discriminator. As the name implies, the generator generates synthetic data, where as the discriminator discerns whether the synthetic data created by the generator is real or fake. The primary objective of the generator is to produce data that closely resembles real data, as judged by the discriminator. Consequently, GANs are predominantly employed in the field of medical imaging for data generation and augmentation [18]. Using GAN for data augmentation is the ultimate approach employed in deep learning to mitigate overfitting and enhance the performance of deep learning models [33]. GANs are used to generate synthetic data that are incorporated into the original dataset, thereby expanding the dataset. This enables the model to train on a larger volume of data, increases the data diversity, and facilitates its ability to generalize across various conditions. Moreover, GANs have been utilized for the restoration of impaired medical images, such as the restoration of partially lost segments in vascular images [34]. In addition, GANs are employed for super-resolution tasks, where a generator is trained to convert low-resolution images into high-resolution images [35]. This study focused on utilizing a generative GAN model to generate denoised images. The goal of GAN training was to teach the generator how to create denoised images by learning from noisy ground truth images. Ultimately, the generator should be capable of producing denoised images when provided with noisy input images. Compared to the conventional filtering method, the filtering method required manual filtering to reduce noise, and it was impossible to estimate the level of noise. However, the advantage of using GAN is that the noise level can be estimated and an automated noise image can be generated to reduce noise. The quantitative evaluation of the denoised images obtained through GAN training revealed improvements with a CNR of 2.80%, COV of 12.50%, and overall NNPS.

However, while the COV and NNPS showed substantial improvements, the enhancement in CNR appeared to lag. The evident improvement in the COV and NNPS suggests a clear improvement in noise handling. Nevertheless, the relatively minor improvement in the CNR implies the narrowing of the gap between the signal and background. This suggests the enhancement of breast tissue signal during the noise removal process. This aspect may find utility as a model capable of increasing the contrast in images, such as computed tomography scans with a black background. However, the further optimization of the network through enhancements in learning rates, parameter tuning, and overall CNR improvement is expected to lead to a more effective noise reduction algorithm in the future. Considering the inherent network characteristics of GANs, which often require considerable time and pose challenges in setting hyperparameters and optimizing the model, it is imperative to seek improvements in these aspects. Additionally, the limited number of acquired breast X-ray images posed challenges in optimizing the network during the training process. Consequently, in future studies, obtaining a larger dataset and implementing data augmentation will likely be necessary to facilitate training. Although this study was conducted exclusively using breast images, it is important to assess the differences and potential variations that may arise when conducting experiments using images of other organs or tissues. Additional data acquisition and training processes are necessary to adapt the model to different anatomical regions because the characteristics and nuances of medical images can vary significantly from one organ or tissue to another.

In this study, we evaluated the image quality using CNR, COV, and NNPS. However, the inclusion of additional metrics such as signal-to-noise ratio (SNR), modulation transfer function (MTF), peak signal-to-noise ratio (PSNR), and structural similarity index measure (SSIM) will be warranted in future studies. SNR serves as a metric indicating the desired signal-to-noise ratio within an image, with higher SNR values signifying improved image quality [36]. MTF measures the extent of signal attenuation in various spatial frequency bands within an image and is used to evaluate image sharpness [37]. PSNR quantifies the difference between the original image and a noise-contaminated image, with higher values indicating reduced noise [38]. Furthermore, SSIM is used to gauge structural similarity between the original image and a noise-reduced image, mimicking human visual system responses [39]. Employing a diverse array of evaluation metrics will enable the development of robust and reliable software for assessing image quality.

In this study, we developed a deep-learning-based algorithm that allows for the estimation of amplified noise levels induced by the use of virtual grids. Consequently, a noise image can be derived at different levels, ultimately leading to the acquisition of noise-reduced images. Tissues with high density and numerous fine structures, such as the breast, are particularly sensitive to noise. Various types of noise in breast X-ray images can obscure subtle signs of breast cancer, making it especially challenging to detect microcalcifications in the range of 0.1–1 mm [9]. Furthermore, the presence of misaligned and distorted pixel values within the images leads to significant degradation in image quality. Therefore, noise reduction in breast X-ray images is of paramount importance. The results of this study are anticipated to enhance the accuracy of early breast cancer detection, which can be challenging for medical experts, by mitigating noise in breast X-ray images.

Advancements in artificial intelligence (AI) have led the healthcare industry to increasingly depend on larger volumes of data. The quantity of data significantly influences the accuracy of AI, which in turn can have a direct impact on diagnosis and treatment outcomes [30]. Therefore, a well-constructed database will lead to a greater synergistic effect between AI and the healthcare sector.

## 5. Conclusions

We aimed to address the issue of noise amplification when using a virtual grid by modeling an algorithm capable of estimating radiation noise at different levels. Subsequently, we developed a GAN-based noise reduction algorithm to enhance the virtual grid software. The effectiveness of noise reduction through GAN-based noise reduction was confirmed through COV and CNR measurements. Additionally, NNPS measurements indicated improvements across all frequency ranges when the algorithm was applied. In conclusion, the application of the GAN-based noise reduction algorithm developed in this study effectively reduced noise, thereby demonstrating the acquisition of improved-quality breast X-ray images.

## Figures and Tables

**Table 1:** Training parameters of the GAN architecture.

Parameter	Dimension
Size of input and output images	256 × 256
Number of training patches	54,686
Number of validating patches	6100
Number of testing patches	2128
Number of epochs	200
Size of batch	16
Number of channels	128, 256, 512, and 1024
Learning rate	5 × 10^−4^
Objective function	Mean Squared Error Loss
Optimization solver	Adaptive momentum estimation (Adam)

## Data Availability

Data are contained within the article.
